# Use of a Luciferase-Expressing Orthotopic Rat Brain Tumor Model to Optimize a Targeted Irradiation Strategy for Efficacy Testing with Temozolomide

**DOI:** 10.3390/cancers12061585

**Published:** 2020-06-15

**Authors:** Alexandra M. Mowday, Natasja G. Lieuwes, Rianne Biemans, Damiënne Marcus, Behzad Rezaeifar, Brigitte Reniers, Frank Verhaegen, Jan Theys, Ludwig J. Dubois

**Affiliations:** 1 The M-Lab, Department of Precision Medicine, GROW—School of Oncology and Developmental Biology, Maastricht University, 6229ER Maastricht, The Netherlands; a.mowday@maastrichtuniversity.nl (A.M.M.); n.lieuwes@maastrichtuniversity.nl (N.G.L.); rianne.biemans@maastrichtuniversity.nl (R.B.); d.marcus@maastrichtuniversity.nl (D.M.); jan.theys@maastrichtuniversity.nl (J.T.); 2 Department of Radiation Oncology (Maastro), GROW—School of Oncology and Developmental Biology, Maastricht University, 6229ER Maastricht, The Netherlands; Behzad.Rezaeifar@maastro.nl (B.R.); frank.verhaegen@maastro.nl (F.V.); 3 Research group NuTeC, Centre for Environmental Sciences, Hasselt University, BE3590 Diepenbeek, Belgium; brigitte.reniers@uhasselt.be

**Keywords:** glioblastoma, orthotopic models, targeted radiotherapy, bioluminescence imaging, CT imaging, temozolomide, standard of care

## Abstract

Glioblastoma multiforme (GBM) is a common and aggressive malignant brain cancer with a mean survival time of approximately 15 months after initial diagnosis. Currently, the standard-of-care (SOC) treatment for this disease consists of radiotherapy (RT) with concomitant and adjuvant temozolomide (TMZ). We sought to develop an orthotopic preclinical model of GBM and to optimize a protocol for non-invasive monitoring of tumor growth, allowing for determination of the efficacy of SOC therapy using a targeted RT strategy combined with TMZ. A strong correlation (r = 0.80) was observed between contrast-enhanced (CE)-CT-based volume quantification and bioluminescent (BLI)-integrated image intensity when monitoring tumor growth, allowing for BLI imaging as a substitute for CE-CT. An optimized parallel-opposed single-angle RT beam plan delivered on average 96% of the expected RT dose (20, 30 or 60 Gy) to the tumor. Normal tissue on the ipsilateral and contralateral sides of the brain were spared 84% and 99% of the expected dose, respectively. An increase in median survival time was demonstrated for all SOC regimens compared to untreated controls (average 5.2 days, *p* < 0.05), but treatment was not curative, suggesting the need for novel treatment options to increase therapeutic efficacy.

## 1. Introduction

Glioblastoma multiforme (GBM) is an aggressive and frequently occurring primary malignant brain cancer with a mean survival time of less than 15 months after initial diagnosis, regardless of treatment [1]. Despite recent advancement in the understanding of the molecular pathogenesis behind the disease and improvement in diagnostic ability, little has changed in terms of prognosis. GBM remains a lethal disease, and most patients (>70%) will die within two years [1,2]. Standard-of-care therapy for newly diagnosed GBM consists of surgical resection, if feasible, and/or regional radiotherapy (RT) with concomitant or adjuvant temozolomide (TMZ), which provides an improvement in median survival of only 2.5 months [1]. Bevacizumab is approved for patients with recurrent GBM, but only prolongs progression-free survival with no impact on overall survival [3].

Until recently, large-field single-beam irradiation was commonplace in preclinical animal studies [4,5]. Radiation dose was crudely estimated and efficacy studies were often hampered by high doses of radiation to healthy tissue. Therefore, tumors were often implanted subcutaneously to enable relatively easy targeting with a single beam and some shielding. Yet subcutaneous glioma models lack the appropriate central nervous system microenvironment and are poorly predictive of therapeutic outcome, particularly for anti-angiogenic drugs or metabolic inhibitors [6]. Orthotopically implanted gliomas in syngeneic, immunocompetent animals are thought to provide the most accurate representation of the biological features of cancer growth and metastasis in humans [7]. Modern techniques now permit closer replication of clinical practice when irradiating orthotopic models, and recently developed treatment planning systems allow for better protection of healthy tissue from the radiation dose [4,5,8,9].

The histopathological characteristics of human GBM include areas of intratumoral hypoxia and necrosis [10], in addition to a diffuse growth infiltrate into the neuropil [11]. Yet, frequently used preclinical GBM models (e.g., C6, 9L, and U87) are generally immunogenic to the host, contain minimal hypoxia, and tumor growth is sharply delineated with little infiltration into normal brain tissue [12,13,14]. In experimental neurooncology, there is thus a strong need for clinically relevant preclinical models that are reproducible and physiologically applicable to the human condition. The F98 rat glioma cell line was originally obtained following administration of ethylnitrosourea to pregnant rats, whose progeny developed brain tumors [15]. Similar to the features of human GBM, these gliomas have an infiltrative pattern of growth, are weakly immunogenic, express relevant cellular markers and have areas of tumor hypoxia with a necrotic core [12,16,17,18], making them a strong candidate for use as an orthotopic GBM model.

The aim of this study was to use the F98 orthotopic rat glioma model to optimize a targeted RT strategy for GBM in a manner which is similar to clinical practice. Development of a RT beam protocol that maximally spared the radiation dose to healthy tissues then allowed for accurate determination of the efficacy of SOC therapy in this model for the first time.

## 2. Results

### 2.1. Orthotopic F98 GBM Tumors Can Be Monitored Non-Invasively by BLI and CE-CT

**Figure 1:**
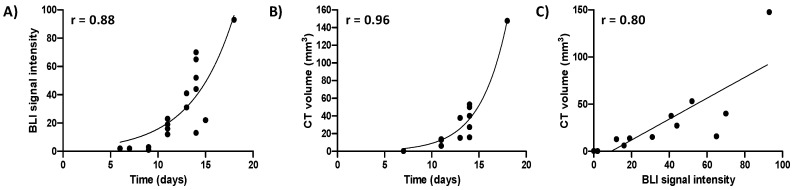
Bioluminescent (BLI) signal intensity and contrast-enhanced (CE)-CT-based tumor volumes are significantly correlated. (**A**) BLI signal intensity over time of *n* = 7 orthotopic F98 luciferase-expressing glioblastoma (GBM) tumors; (**B**) CE-CT-derived tumor volume measurements over time of *n* = 6 orthotopic F98 luciferase-expressing GBM tumors; (**C**) correlation between CE-CT-derived tumor volume and BLI signal intensity for *n* = 6 animals.

In order to monitor tumor growth and treatment efficacy non-invasively, F98 glioma cells were engineered to overexpress firefly luciferase, permitting bioluminescent imaging (BLI) of tumor cells [19]. To allow longitudinal treatment monitoring without cumulative radiation dose, we investigated whether BLI-based signal intensity could substitute for CE-CT-based tumor volume assessment. BLI signal was observed and localized to the tumor site and CE-CT confirmed that BLI signal was related to tumor growth (Figure S1A,B). Both increased exponentially over time (r = 0.88 and 0.96 respectively, Figure 1A,B), and the bioluminescent signal correlated significantly with the tumor volume assessed by CE-CT imaging (r = 0.80, *p* ≤ 0.01, *n* = 12, Figure 1C). This correlation allowed for BLI-based signal intensity to be used as a substitute for CE-CT imaging.

### 2.2. Radiation Treatment Planning Allows for Precise Tumor Targeting and Maximal Sparing of Healthy Tissues

**Figure 2:**
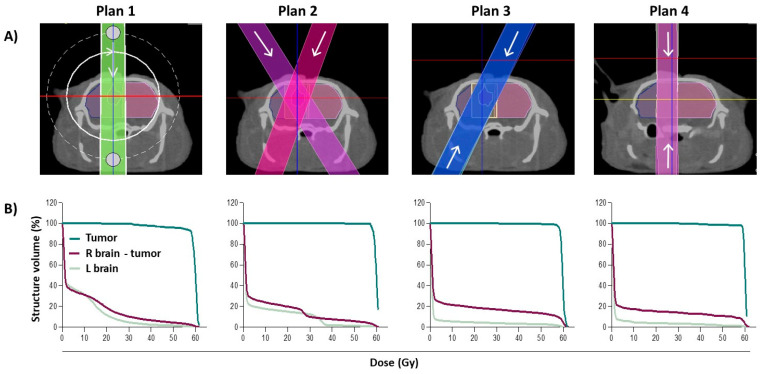
Radiation treatment planning. (**A**) Representative beam set-up in the axial plane for four radiation treatment plans: Plan 1—360° arc; Plan 2—two angled beams; Plan 3—two angled beams, parallel opposed; Plan 4—anterior/posterior opposed (AP-PA). (**B**) The corresponding dose–volume histograms for each radiation treatment plan. R brain = right brain; L brain = left brain.

In order to optimize tumor targeting with maximal sparing of organs at risk (OARs) during radiation treatment for SOC therapy, four different radiation plans were evaluated using a planned dose of 60 Gy as an example. The resulting dose–volume histograms (DVHs) and dose metrics of the tumor and key OARs for each of the in vivo radiation treatment plan options is shown in Figure 2 and Table S1, respectively. The dose–volume metrics indicate that there is no significant difference in the mean dose delivered to the tumor using each of the plans (*p* = 0.54); all plans deliver ≥99% of the expected dose to the tumor area. The uniformity of the dose (as measured by the dose to the hottest 5% (D5) and 95% (D95) of the tumor volume) was also very similar across all four treatment plans, ranging from 102 to 103% and 95 to 98% for average D5 and D95, respectively.

To assess normal tissue sparing, we evaluated the dose delivered to the ipsilateral side of the brain (not including the tumor) and the contralateral side of the brain to the tumor site. On the ipsilateral side, there was no significant difference in mean dose delivered to this area (*p* = 0.07), with all treatment plans sparing this side of the brain ≥80% of the planned dose. However, on the contralateral side of the brain, Plan 3 and 4 received a significantly reduced mean dose when compared to Plan 1 and 2 (*p* ≤ 0.001), improving normal tissue sparing on this side of the brain from 92 to 97%. For this reason, Plan 1 and 2 were immediately eliminated as possible radiation plan options. Although there was no significant difference in the mean dose received on the contralateral side between Plan 3 and 4 (1.8 and 1.6 Gy, respectively *p* = 0.76), when looking into the uniformity of the delivered dose (minimum dose to the hottest 5% (D5) and 95% (D95) of the area volume), the D5 value for Plan 4 was moderately lower than for Plan 3, suggesting improved tissue sparing. The AP-PA plan (Plan 4) was therefore selected for use in the SOC efficacy experiments.

### 2.3. SOC Treatment Using a Targeted Radiation Strategy Demonstrates Anti-Tumor Efficacy but Is Not Curative

**Figure 3:**
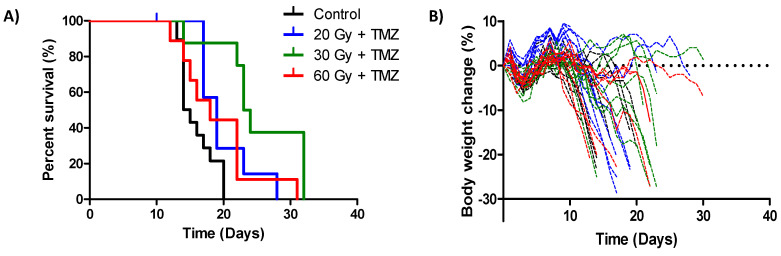
Therapeutic efficacy of standard-of-care (SOC) therapy using a targeted radiation strategy (**A**) Kaplan–Meier survival curves of animals following orthotopic F98 GBM implantation and a single dose of targeted radiation (as specified) with concomitant temozolomide (TMZ). Survival endpoint was defined as the time an animal left the experiment for humane reasons. *n* ≥ 8 animals per treatment group. (**B**) Body weight loss of the treatment groups over time following surgical implantation of the tumor. Percentage body weight change relative to starting body weight was determined.

Optimization of the radiation treatment plan allowed for subsequent testing of SOC therapy in this model. A dose response of radiation from 0 to 60 Gy was used in an attempt to determine the dose of radiation that locally controls 50% of the tumors (TCD50). An increase in median survival time was demonstrated for all SOC regimens compared to untreated control animals (on average 5.2 days, *p* < 0.05, log-rank test), but this was not curative for any animals (Figure 3A). Surprisingly, there was no relationship between the increase in radiation dose and median survival time. Animals treated with 30 Gy received the largest survival benefit (23.5 days) in comparison to the animals treated with 20 or 60 Gy (19 and 18 days, respectively). In total, 75% of the animals treated with 60 Gy demonstrated ≥10% body weight loss, compared to 63% for the 20 and 30 Gy treatment groups (Figure 3B). In addition, the body weight loss in the 60 Gy group occurred at an earlier time point on average than in the 30 Gy group (day 16 and 18, respectively), after which there was a steady decline to humane endpoint with no recovery.

Radiation dose metrics of the treated animals were analogous to those generated in Plan 4 (AP-PA) during the optimization phase. On average across all treatment groups, the tumor received 96.4% of the expected dose (Table 1). The ipsilateral side of the brain (not including the tumor) was spared on average 84% of the dose. In fact, 95% of this area received ≤0.2 Gy, regardless of the administered dose. The remaining 5% of the area was not spared. However, due to the angle of the beams, this was not unexpected. The contralateral side of the brain was even further spared from radiation (by an average of 99% of the dose), thus illustrating our targeted radiation strategy.

## 3. Discussion

Modern techniques have allowed for closer replication of human radiotherapy practice; fractionated therapy is now possible with multiple, conformal beams and sophisticated dose calculation algorithms. However, these methods require high-resolution imaging techniques to allow for precise and accurate beam positioning, particularly when working with orthotopic tumor models such as GBM. CT is the preferred imaging modality for the target delineation and treatment planning of solid tumors using external beam radiation therapy as it most closely mimics clinical practice [20]. Here, a CT-based treatment planning system (SmART-ATP) allowed for delivery of a highly localized prescribed dose to an isocenter placed at the center of the target (tumor). Dose calculations performed using this set-up result in greater dose homogeneity and minimization of the dose received by the OARs [4,5,8,9].

The AP-PA beam plan was chosen for the SOC efficacy study as this plan maximized normal tissue sparing. While this plan accurately delivered the proposed radiation dose to the tumor (99% of the planned mean dose was delivered to the experimental tumors), the normal tissue sparing in the experiment was slightly greater than expected, thus exemplifying our targeted irradiation strategy. The ipsilateral side of the brain without the tumor was expected to be spared 82% of the planned dose (49 Gy), but was in fact spared 84% (50 Gy). In addition, the minimum dose for the hottest 5% of this area (D5) was reduced from 59.8 Gy to just 52.9 Gy.

It is possible to use other methods for treatment planning, such as MRI, which is generally considered the standard imaging modality for preclinical intracranial tumors. Previous reports suggest that MRI and CT-derived GBM tumor volume measurements show a strong correlation in vivo [21]. However, others have reported that CT was not sufficient to achieve accurate irradiation of the target in a GBM model (although this was performed without contrast) [22]. MRI was required in the study mentioned to deliver a homogeneous dose using a more complicated arrangement of three non-coplanar arcs. Regardless, in the absence of robust radiation treatment planning software for MRI, additional CT scans are still required to perform dose calculations, thereby limiting the utility of this approach [22,23,24].

Optimization of the radiation treatment plan allowed for subsequent SOC efficacy testing. Although each treatment group individually provided significant additional efficacy when compared to untreated controls, there was no improvement in efficacy with regard to the radiation dose-response tested. Animals in the 30 Gy treatment group received the largest therapeutic benefit and the TCD50 was unable to be determined. This can be explained in part by the body weight loss observed following treatment. Animals in the 60 Gy group suffered from earlier and more severe body weight loss, which had a major impact on overall survival. It is unknown whether this is occurring due to radiation toxicity or tumor progression, although it is likely to be a combination of the two. Others have successfully delivered a single fraction of 60 Gy to rats bearing human GBM, with animals surviving for at least ten weeks after treatment [25]. However, only a portion of the tumor received 60 Gy in the study mentioned, resulting in severe necrosis in these areas of the tumor [25]. The clinical symptoms of radiation-induced necrosis include worsening neurologic signs and symptoms and neurocognitive decline [26], which when extrapolated to whole tumor irradiation could possibly explain the toxicity observed in our study. Standard radiotherapy for patients with GBM is a fractionated schedule of 2 Gy per day, five days per week, for six weeks for a total of 60 Gy [1]. However, the approximate relative biological effectiveness of this is likely to be closer to 20 Gy [22]. A hypofractionated schedule might potentially reduce toxicity and improve efficacy in the context of our study (e.g., six fractions of 10 Gy), as hypofractionated radiotherapy schemes have been successfully utilized in patients to improve convenience and tolerance to therapy, particularly in the elderly, where treatment-related toxicities are a main concern [27,28].

Similar to what is observed in patients, while SOC was therapeutically effective, it was not curative, suggestive again of the clinical relevance of F98 to human GBM. Extensive tumor hypoxia, necrosis, and an infiltrative-like growth pattern of irregular tumor borders and peripheral extension into the surrounding tissue was demonstrated in F98 tumors (Figure S1C,D) and is also frequent in human GBM, being negative prognostic indicators associated with biological and clinical aggressiveness, shorter time to tumor recurrence, and reduced survival [29,30]. Hypoxic tumor cells are genetically unstable and show increased expression of O6-methylguanine-DNA-methyltransferase (MGMT) expression, a DNA repair enzyme known to negate TMZ-induced DNA alkylation [31]. Thus, tumor hypoxia is thought to confer resistance to TMZ chemotherapy. Targeting of these hypoxic cells could therefore increase the therapeutic efficacy of TMZ treatment. Hypoxia-activated prodrugs (HAPs) are selectively activated by enzymatic reduction in hypoxic cells, and may provide a means to test this hypothesis. One of the most clinically advanced HAPs, evofosfamide, has successfully demonstrated efficacy towards glioblastoma in a preclinical rodent model [32] and in human patients [33] but has yet to be combined with SOC. HAP administration prior to SOC therapy could potentially remove both the TMZ-resistant and radio-resistant hypoxic cells, providing additional benefit to both components of SOC.

Immune suppression also plays an important role in GBM progression through a variety of mechanisms, including recruitment of M2-associated macrophages to the tumor microenvironment [34] and expression of potent immunosuppressive factors including TGF-β (transforming growth factor beta) and PD-L1 (programmed death-ligand 1) [35]. In addition, TMZ can induce lymphopenia in malignant glioma patients treated with SOC therapy [36]. Combining SOC with an immune-stimulating approach could therefore be advantageous. However, recently developed cancer immunotherapies have had disappointing results in this disease setting, likely due to the fact that only a single component of the anti-tumor immune response is targeted [37]. Tumor-targeting bacteria that can cross the blood–brain barrier such as *Clostridium* could be used to provide an inflammatory payload exquisitely to the tumor microenvironment, potentially combining the innate immune response to infection with effective stimulation of immune memory against the tumor [38]. Overall, our therapeutic results suggest that a combination of treatment modalities with SOC will be required to improve therapeutic outcome.

In this study, we also demonstrate a significant correlation between bioluminescent signal intensity and tumor volume assessed by CE-CT in the F98 model. Similar correlations (r > 0.54) have previously been reported for orthotopic mouse GBM in mice [20,39]. However, these studies were performed using a human primary GBM cell line that lacks key features of clinical GBM [13]. Importantly, the correlation observed in this study suggests that BLI can be used as a surrogate for CE-CT, reducing the radiation burden of using frequent CE-CT scans for long-term treatment monitoring and providing an integrated platform for GBM evaluation.

## 4. Materials and Methods

### 4.1. Generation of the F98 Luciferase-Expressing Cell Line

F98 GBM cells (kindly provided by Prof. C. Vanhove, Ghent University, Belgium) were cultured in DMEM (Dulbecco’s modified eagle medium) supplemented with 10% fetal bovine serum (Sigma-Aldrich, Zwijndrecht, Netherlands). Cells were transduced with a lentiviral vector harboring a PGK-driven FLuc+ (pLenti PGK V5-LUC neo (w623-2), a gift from Eric Campeau (Addgene plasmid #21471; http://n2t.net/addgene:21471; RRID:Addgene_21471)) [40]. Neomycin selection began 48 h after transduction and after three weeks of continuous selection, cells could be used in experiments. Cells were tested for the presence of mycoplasma prior to injection into animals.

### 4.2. Orthotopic GBM Tumor Implantation

Implantation of the orthotopic GBM tumors was performed as previously described [22], with some modifications. Briefly, young (8–12 weeks) female F344/IcoCrl rats were ordered from Charles River (‘s-Hertogenbosch, Netherlands). Then, 30 min prior to surgery, animals were administered analgesia by intraperitoneal injection (buprenorphine, 0.05 mg/kg). Following sedation (first 4% isoflurane in an induction box, then 2.5% for maintenance), each rat was placed in a stereotaxic frame and the top of the skull was shaved and disinfected with isobetadine. A small incision was made through the skin along the length of the middle of the skull and connective tissue was removed using a sterile cotton bud, before the area was treated with lidocaine (1–2%). A burr was used to drill a hole in the skull 2 mm caudal and 2.5 mm right from bregma. In total, 20,000 cells (in 5 µL of phosphate buffered saline (PBS)) were slowly (2 µL per minute) injected at a depth of 3 mm. The needle was then left for 5 min to ensure no reflux of cells. The drill hole was closed with bone wax (Aesculap AG^®^, B.Braun, Melsungen, Germany) and non-absorbable sutures (Ethicon, Johnson & Johnson, Amersfoort, Netherlands) were used to close the incision. Post-operative analgesia was given as required (carprofen, 2–4 mg/kg in the drinking water). All animal experiments were in accordance with local institutional guidelines for animal welfare and approved by the Animal Ethical Committee of the University of Maastricht (Protocol # 2017-012).

### 4.3. Microscopy

Excised rat brains were fixed in neutral-buffered formalin (4%) before embedding in paraffin wax. Sections (7 µm) were cut, mounted onto poly-L-lysine-coated slides, and heat-fixed for 30 min at 58 °C. Paraffin was removed and sections were rehydrated using sequential immersions in 100% xylene, 100% ethanol, 80% ethanol, 50% ethanol and 100% distilled water. At this point, hematoxylin and eosin (H&E) staining was performed. Following an additional antigen retrieval step, staining for hypoxia was performed using pimonidazole in accordance with the manufacturer’s instructions (Hypoxyprobe™-1 Omni Kit, Hypoxyprobe, Burlington, MA, USA). All images were acquired on a M8 Microscope and Scanner (Precipoint, Freising, Germany).

### 4.4. Bioluminescence Imaging and Analysis

Under isoflurane anesthesia, whole-body white light and BLI scans from a dorsal position were acquired using an iXon Ultra 897 camera (Andor Technology Ltd., Belfast, United Kingdom) in the X-Rad 225Cx machine (Precision X-ray, Inc, North Branford, CT, USA) using no filters (open modus), ten minutes after intraperitoneal injection of D-luciferin (150 mg/kg, Perkin Elmer, Rotterdam, Netherlands). BLI images were acquired with a gain of 5 and an exposure time of 0.005 s (white light) or 60 s (BLI). Signal intensity parameters were consistent for all images (either white light or BLI). The cumulative raw BLI intensity signal was corrected with the background signal corresponding to an area on the rat skin distant from the skull.

### 4.5. Contrast-Enhanced CT (CE-CT) Imaging and Analysis

Under isoflurane anesthesia, CT images of the skull area were acquired using an X-Rad 225Cx small-animal irradiator (Precision X-Ray, Inc) immediately following intravenous injection of contrast-enhancing agent (60 mg/kg Omnipaque, GE Healthcare, Eindhoven, Netherlands). CT acquisition parameters, CT number to density calibration and medium segmentation, were performed as described previously [41]. The CT imaging dose was 30 cGy. Images were reconstructed using Feldkamp’s filtered back-projection (Pilot version 1.14.4, Precision X-Ray, Inc).

### 4.6. Radiation Treatment Planning and Dose Calculation

Treatment plans based on 1) 360° arc, 2) two angled beams (15°/345°), 3) two angled beams, parallel opposed (15°/195°) and 4) anterior/posterior opposed (AP-PA, 0°/180°) using a 5 mm circular collimator (SmART-ATP version 2.0, Smart Scientific Solutions B.V., Maastricht, Netherlands) were compared for mean dose (Dmean), dose to 95% (D95) and dose to 5% (D5) of the CT-delineated tumor volume. Dose calculations were performed with the Monte Carlo dose engine DOSXYZnrc (National Research Council Canada) using an intrinsic dose uncertainty set to 5% in the target volume. Radiation delivery was performed according to ACROP guidelines as previously described [42,43]. Briefly, irradiations were performed at 225 kVp and 13 mA, with an inherit 0.8 mm beryllium filter and an additional 0.3 mm copper filter, resulting in a spectrum with a half value layer of 0.98 mm copper. The 5 mm beams had a full-width half maximum of 4.9 mm and a penumbra size of 0.5 mm (20–80% of maximum dose) at the isocenter and a dose rate of approximately 2.5 Gy/min at the source-to-isocenter distance of 303.6 mm. All calculated dose values were scored as dose-to-medium, transport-in-medium.

### 4.7. Tumor Growth Delay

Tumor-bearing rats were randomized to treatment groups after second positive BLI and first positive CT signal and irradiated with 0, 20, 30 or 60 Gy. Concomitant temozolomide (29 mg/kg, Bioconnect, Huissen, Netherlands) was administered by intraperitoneal injection daily for four days, beginning on the day of irradiation. Tumor growth was monitored 3× per week using BLI and body weight was monitored daily. BLI-based tumor volume was determined by signal intensity and CT-based tumor volume was calculated using a summation of all planes. Animals were sacrificed if body weight loss exceeded 20% of the pretreatment value or if neurological symptoms were observed (humane endpoint). Kaplan–Meier plots were constructed to calculate median time to survival endpoint, defined as the time an animal left the experiment due to humane endpoint (clinical neurological symptoms and/or body weight loss, indicative of tumor progression). Treatment efficacy was assessed by comparing the median survival time with untreated control animals.

### 4.8. Statistical Analysis

Statistical analyses (one-way ANOVA, Student’s *T*-test, log-rank test) were performed using GraphPad Prism Software (version 5.03). A *p*-value < 0.05 was considered statistically significant.

## 5. Conclusions

In summary, optimization of a targeted RT treatment strategy for GBM resulted in maximum sparing of healthy brain tissue while ensuring accurate delivery of ≥99% of the dose to the tumor area. However, it appears that truly effective GBM therapy will require a combination of treatment modalities with SOC to increase therapeutic efficacy and improve clinical outcome. Use of a clinically relevant orthotopic model with multimodal imaging capability such as this will be essential for preclinical development in this context.

## Figures and Tables

**Table 1:** Dose–volume histogram metrics for different tissue structures (tumor, right brain (R brain) without tumor, and left brain (L brain)) in treated animals (20, 30 or 60 Gy as indicated) using the AP-PA radiation treatment plan (Plan 4). Numbers are the mean and standard deviation of *n* ≥ 7 animals per treatment group. D95 = dose to 95% of the target volume; D5 = dose to 5% of the target volume.

Planned Dose (Gy)	Tumor Dose (Gy)			R Brain without Tumor Dose (Gy)			L Brain		
	Mean	D95	D5	Mean	D95	D5	Mean	D95	D5
20 Gy	19.4 ± 0.3	16.8 ± 0.2	22.2 ± 0.3	3.8 ± 0.3	0.00	20.0 ± 0.4	0.11 ± 0.1	0.00	0.09 ± 0.1
30 Gy	28.1 ± 3.0	21.3 ± 11	30.4 ± 0.3	4.1 ± 1.5	0.08 ± 0.0	26.1 ± 7.6	0.58 ± 0.4	0.07 ± 0.1	1.1 ± 1.0
60 Gy	59.2 ± 1.5	54.1 ± 9.4	61.0 ± 0.4	9.3 ± 3.8	0.22 ± 0.1	52.9 ± 19	1.2 ± 0.9	0.19 ± 0.1	3.0 ± 4

## Supplementary Materials

The following are available online at https://www.mdpi.com/2072-6694/12/6/1585/s1, Figure S1: F98 tumor growth over time as determined by non-invasive BLI and CE-CT imaging; Table S1: Dose–volume histogram metrics for different tissue structures using the four different radiation plans described in Figure 3.
