# Supplementary material for: Use of a Luciferase-Expressing Orthotopic Rat Brain Tumor Model to Optimize a Targeted Irradiation Strategy for Efficacy Testing with Temozolomide
Source: Cancers (Basel). 2020 Jun 15;12(6):1585. doi: 10.3390/cancers12061585 (PMC7352586; doi:10.3390/cancers12061585)
Supplement: Supplementary file 1 [file cancers-12-01585-s001.pdf]

cancers

Article

Use of a Luciferase-Expressing Orthotopic Rat Brain Tumor Model to Optimize a Targeted Irradiation Strategy for Efficacy Testing with Temozolomide

Alexandra M. Mowday, Natasja G. Lieuwes, Rianne Biemans, Damiënne Marcus, Behzad Rezaeifar, Brigitte Reniers, Frank Verhaegen, Jan Theys and Ludwig J. Dubois

Supplementary Materials

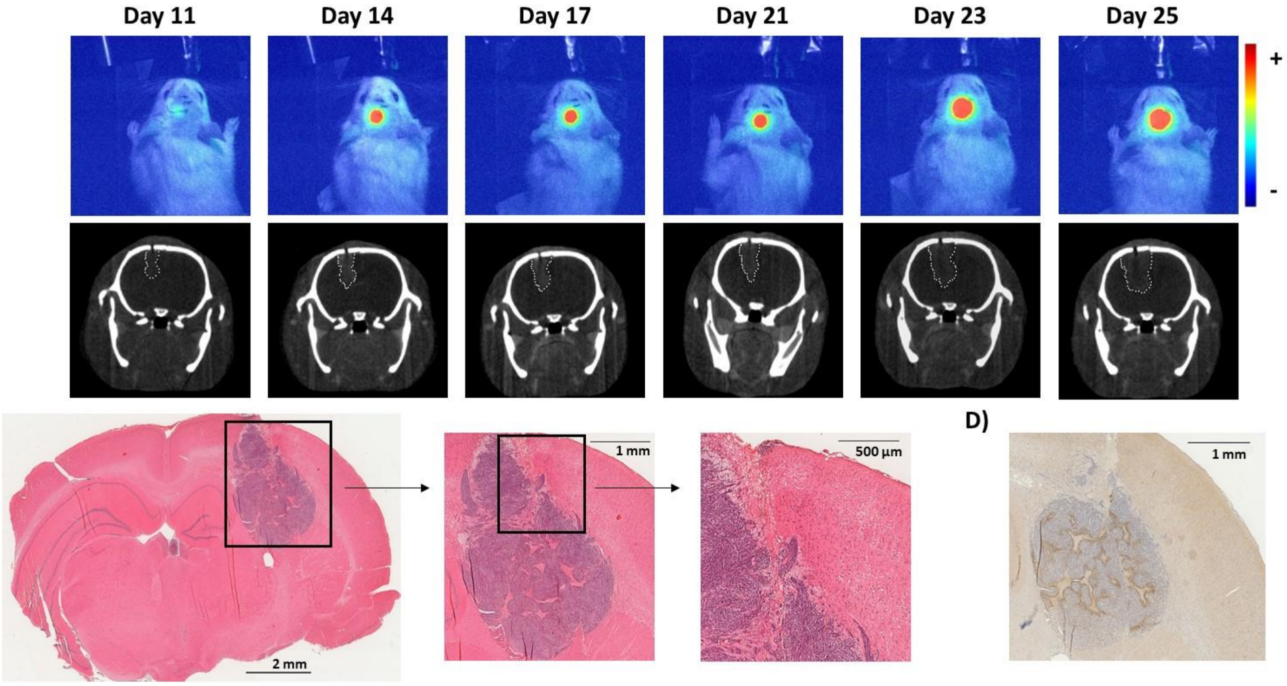

Figure S1. F98 tumor growth over time as determined by non-invasive BLI and CE-CT imaging. (**A**) Representative 2D BLI images overlaid over the white light image of the animal and (**B**) The corresponding CE-CT images of the same animal (tumor delineated in white). Both image sets are at selected time points after surgical implantation of F98 cells in the brain. Representative (**C**) H&E and (**D**) Pimonidazole (hypoxia) GBM image.

Table S1. Dose-volume histogram metrics for different tissue structures (tumour, right brain (R brain) without tumour, left brain (L brain)) using the four different radiation plans described in Figure 3. Prescribed dose was 60 Gy. Numbers are the mean and standard deviation of *n* = 4 individual animals bearing an orthotopic F98 GBM. D95 = dose to 95% of the target volume, D5 = dose to 5% of the target volume.

Plan	Tumour Dose (Gy)			R Brain Without Tumor Dose (Gy)			L Brain Dose (Gy)		
	**Mean**	**D95**	**D5**	**Mean**	**D95**	**D5**	**Mean**	**D95**	**D5**
Plan 1	59.5 ± 0.5	57.1± 2.1	61.2 ± 0.2	10.3 ± 0.7	0.30 ± 0.00	52.6 ± 3.5	6.2 ± 0.8	0.25 ± 0.05	24.3 ± 3.0
Plan 2	59.8 ± 0.2	58.2 ± 0.4	61.6 ± 0.7	10.4 ± 1.0	0.25 ± 0.05	56.1± 2.1	3.7 ± 1.7	0.23 ± 0.04	24 .3 ± 8.9
Plan 3	59.7 ± 0.3	58.6 ± 0.4	60.9 ± 0.2	12.3 ± 0.9	0.30 ± 0.00	60.0 ± 0.3	1.8 ± 0.9	0.23 ± 0.04	5.0 ± 6.3
Plan 4	59.9 ± 0.1	57.1± 0.1	61.2 ± 0.3	11.0 ± 1.3	0.25 ± 0.05	59.8 ± 0.4	1.6 ± 0.7	0.23 ± 0.04	3.5 ± 3.2
